# Engineering acetyl-CoA supply and *ERG9* repression to enhance mevalonate production in *Saccharomyces cerevisiae*

**DOI:** 10.1093/jimb/kuab050

**Published:** 2021-08-05

**Authors:** Scott A Wegner, Jhong-Min Chen, Samantha S Ip, Yanfei Zhang, Deepak Dugar, José L Avalos

**Affiliations:** Department of Molecular Biology, Princeton University, Princeton, NJ 08544, USA; Department of Chemical and Biological Engineering, Princeton University, Princeton, NJ 08544, USA; Department of Chemical and Biological Engineering, Princeton University, Princeton, NJ 08544, USA; Department of Chemical and Biological Engineering, Princeton University, Princeton, NJ 08544, USA; Visolis, Inc., 1488 Zephyr Ave. Hayward, CA 94544, USA; Department of Molecular Biology, Princeton University, Princeton, NJ 08544, USA; Department of Chemical and Biological Engineering, Princeton University, Princeton, NJ 08544, USA; The Andlinger Center for Energy and the Environment, Princeton University, Princeton, NJ 08544, USA; High Meadows Environmental Institute, Princeton University, Princeton, NJ 08544, USA

**Keywords:** Metabolic engineering, Mevalonate, Acetyl-CoA, *Saccharomyces cerevisiae*, *ERG9*, Pantothenate

## Abstract

Mevalonate is a key precursor in isoprenoid biosynthesis and a promising commodity chemical. Although mevalonate is a native metabolite in *Saccharomyces cerevisiae*, its production is challenged by the relatively low flux toward acetyl-CoA in this yeast. In this study we explore different approaches to increase acetyl-CoA supply in *S. cerevisiae* to boost mevalonate production. Stable integration of a feedback-insensitive acetyl-CoA synthetase (*Se-acs^L641P^*) from *Salmonella enterica* and the mevalonate pathway from *Enterococcus faecalis* results in the production of 1,390 ± 10 mg/l of mevalonate from glucose. While bifid shunt enzymes failed to improve titers in high-producing strains, inhibition of squalene synthase (*ERG9*) results in a significant enhancement. Finally, increasing coenzyme A (CoA) biosynthesis by overexpression of pantothenate kinase (*CAB1*) and pantothenate supplementation further increased production to 3,830 ± 120 mg/l. Using strains that combine these strategies in lab-scale bioreactors results in the production of 13.3 ± 0.5 g/l, which is ∼360-fold higher than previously reported mevalonate titers in yeast. This study demonstrates the feasibility of engineering *S. cerevisiae* for high-level mevalonate production.

## Introduction

Rising environmental concerns have motivated research in microbial fermentations for chemical production from renewable feedstocks (Jullesson et al., 2015). Metabolic engineering has successfully harnessed the mevalonate pathway to produce a variety of isoprenoids with diverse applications, including as biofuels, fragrances, and pharmaceuticals (Paddon & Keasling, 2014; Vickers et al., 2017). However, fewer studies have focused on the production of mevalonate, a key precursor to isopentenyl pyrophosphate (IPP) and dimethylallyl pyrophosphate (DMAPP), the basic building blocks for all isoprenoids and steroids. As a metabolite that can be readily secreted, mevalonate is a promising product itself for the sustainable production of various polymers derived from mevalonolactone, β-methyl-δ-valerolactone, and anhydromevalonolactone (Ball-Jones et al., 2010; Dugar & Friedberger, 2015; Xiong et al., 2014).

**Fig. 1:**
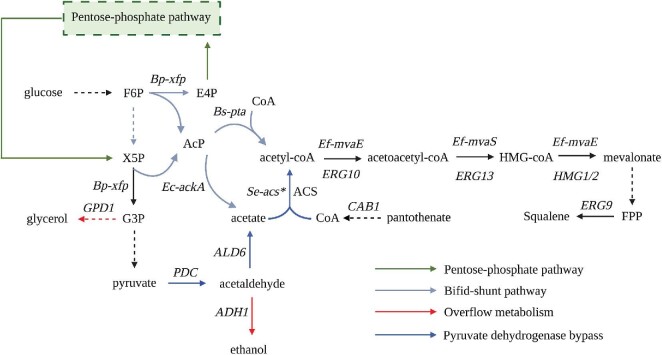
Overview of metabolic pathways to supply acetyl-CoA to mevalonate biosynthesis. See main text for enzyme names associated with each gene. Solid arrows represent specific enzymatic steps, while dashed arrows represent multiple enzymatic reactions. Se-acs* represents the L641P feedback inhibition-resistant acetyl-CoA synthetase from *Salmonella enterica*. Metabolite abbreviations: acetyl-phosphate (AcP), coenzyme A (CoA), D-erythrose 4-phosphate (E4P), D-fructose 6-phosphate (F6P), D-glyceraldehyde 3-phosphate (G3P), 3-hydroxy-3-methylglutaryl coenzyme A (HMG-CoA), D-xylulose 5-phosphate (X5P), farnesyl pyrophosphate (FPP).

*Saccharomyces cerevisiae* is a major industrial organism used in the production of biofuels and chemicals, including isoprenoids, making it an attractive platform for mevalonate production (Vickers et al., 2017). Furthermore, *S. cerevisiae* contains an endogenous mevalonate biosynthetic pathway starting from acetyl-CoA and proceeding in three enzymatic steps (Fig. 1). Acetyl-CoA is first converted by Erg10p (acetoacetyl-CoA thiolase) to acetoacetyl-CoA, which is then converted to 3-hydroxy-3-methyglutaryl-CoA (HMG-CoA) by Erg13p (HMG-CoA synthase). HMG-CoA is then converted to mevalonate by HMG-CoA reductases encoded by *HMG1* and *HMG2*, which are rate-limiting and thus commonly overexpressed in strains engineered to produce isoprenoids (Basson et al., 1986; Ignea et al., 2011; Ro et al., 2006). The highest titers reported in an *S. cerevisiae* strain engineered to make mevalonate as end product came from a study in which cytosolic levels of acetyl-CoA were increased by diverting the tricarboxylic acid cycle, deleting isocitrate dehydrogenase (*IDH1*), and expressing a heterologous ATP citrate lyase (*ACL*) in a strain expressing *Ef-mvaE* and *Ef-mvaS* from the mevalonate biosynthetic pathway of *Enterococcus faecalis* (Rodriguez et al., 2016). However, the relatively modest titers achieved by this approach (approximately 35 mg/l) suggest that there is much room for improvement.

A main challenge of engineering mevalonate production in *S. cerevisiae* is the relatively low flux toward cytosolic acetyl-CoA. This limitation is largely due to the strong drive in this yeast to convert sugars to the overflow metabolites ethanol and glycerol at the expense of biomass and chemical production (De Deken, 1966). Perturbing overflow metabolism, by deletion of *ADH1/4* and *GPD1/2*, has been shown to increase the availability of acetyl-CoA as measured by an increase in the production of the acetyl-CoA-derived product n-butanol (Lian et al., 2014). Another strategy to boost acetyl-CoA production in *S. cerevisiae* is to increase the pull through the pyruvate dehydrogenase (PDH) bypass (Fig. 1). This involves overexpressing acetyl-CoA synthetase (*acs)* alongside acetaldehyde dehydrogenase (encoded by *ALD6)* to promote the conversion of acetate to acetyl-CoA (Shiba et al., 2007). Overexpression of a feedback-insensitive *acs* from *Salmonella enterica (Se-acs^L641P^)* has been shown to be particularly effective at increasing the production of several isoprenoids, as well as n-butanol and naringenin (Chen et al., 2013; Krivoruchko et al., 2013; Lian et al., 2014; Shiba et al., 2007). Another approach to boost acetyl-CoA production is to enhance the biosynthesis of coenzyme A (CoA), which is limited by the conversion of pantothenate to 4-phosphopantothenate catalyzed by pantothenate kinase (*CAB1*, Sandoval et al., 2014). Previous work has demonstrated that *CAB1* overexpression is sufficient to increase acetyl-CoA production (Fig. 1), which can be further enhanced by supplementing pantothenate in the medium (Liu et al., 2017; Schadeweg & Boles, 2006). Thus, several strategies exist for improving acetyl-CoA production through reducing overflow metabolism, engineering the PDH bypass, and increasing CoA supplies.

Heterologous expression of bifid shunt enzymes has also been used to increase acetyl-CoA production in *S. cerevisiae*. This shunt enables a carbon-conserving route to acetyl-CoA through a fructose-6-phosphate/xylulose-5-phosphate phosphoketolase (*xfp*) that produces acetyl-phosphate, which can be converted into acetate through acetate kinase (*ackA*; or through the endogenous enzymes *GPP1/2*) or directly into acetyl-CoA through the activity of a phosphotransacetylase (*pta*; Fig. 1, Bogorad et al., 2013). The bifid shunt enzymes have been shown to be effective in *S. cerevisiae* for improving acetate production and xylose consumption (Papini et al., 2012; Sonderegger et al., 2004). Furthermore, application of the shunt has been successfully applied to produce polyhydroxybutyrate, farnesene, and carotenoids in yeast (Kocharin et al., 2013; Meadows et al., 2016; Su et al., 2020). While other strategies exist to enhance acetyl-CoA production in *S. cerevisiae*, the bifid shunt pathway, along with the PDH bypass, are two prominent strategies to achieve this goal (van Rossum et al., 2016).

Here we apply previous strategies to enhance acetyl-CoA availability for the production of mevalonate in *S. cerevisiae*. First, we combine the PDH bypass with redirection of overflow metabolism. Additionally, we explore the effects of combining the bifid shunt or enhanced CoA synthesis with the PDH bypass. Finally, we examine the improvement in mevalonate production due to downstream pathway repression by downregulating *ERG9*. Through these efforts we demonstrate the viability of *S. cerevisiae* for high-level production of mevalonate.

## Materials and Methods

### Yeast Medium and Chemicals

Standard YPD and synthetic complete (SC) media were used for transformation and propagation of all yeast strains used in this study. SC-Ura or SC-Ura-Trp media were used where appropriate to ensure propagation of 2μ plasmids, while SC-Ura-His was used for CRISPR-mediated integration of the pMET3 promoter into the endogenous *ERG9* locus. Finally, SC + 2 mM methionine was used to repress the pMET3::*ERG9* containing strains. Synthetic complete medium was composed of 3 g Difco yeast nitrogen base without amino acids and ammonium sulfate, 10 g ammonium sulfate, 400 μM inositol, and 4 g amino acid powder mix per liter. Zeocin (1,600 μg/ml) was added to YPD medium for δ-integration. For gene deletion, SC-dropout medium containing L-glutamic acid monosodium as a substitute nitrogen source was supplemented with the appropriate antibiotic. Antibiotic concentrations used to select for gene deletion were 200 μg/ml G418 (Geneticin^®^), 200 μg/ml hygromycin B, or 100 μg/ml nourseothricin (ClonNat^®^). (±)-Mevalonolactone (97%) was used as an analytical standard for mevalonate and was purchased from ACROS Organics. All the chemicals, unless otherwise specified, were purchased from Sigma-Aldrich (St. Louis, MO, USA) or Thermo Fisher Scientific *Inc*. (Waltham, MA, USA).

### Plasmid Construction

All 2μ and integration plasmids were constructed using restriction enzyme double digestion and ligation of genes of interest into the pJLA vector series (Avalos et al., 2013). These vectors allow insertion of multiple gene expression cassettes into a single plasmid, in tandem or inverted directions. The pJLA series also featured the *AmpR* gene for *Escherichia coli* selection on Ampicillin plates, and either *URA3* or *TRP1* markers for *S. cerevisiae* selection in dropout media. The plasmids pJLA121 (with *URA3* marker) and pJLA123 (with *TRP1* marker) were used to construct single-gene plasmids before assembling multiple cassettes together. Plasmids used to integrate gene constructs into δ-sites were constructed in the same way described above using pYZ23 (Zhao et al., 2018). Table 1 describes all plasmids used in the present study.

**Table 1.** Plasmids Used in This Study

Plasmid Name	Description	Source
pVSLS19	2μ plasmid, URA3 marker, P_GPD1_-*Ef-mvaE*-T_ADH1_ P_TEF1_-*Ef-mvaS*-T_ACT1_	This study
pVSLS22	2μ plasmid, URA3 marker, P_GPD1_-*Ef-mvaE*-T_ADH1_ P_TEF1_-*Ef-mvaS*-T_ACT1_ P_PGK1_-*Se-acs^L641P^*-T_CYC1_	This study
pVSLS32	2μ plasmid, URA3 marker, P_GPD1_-*Bp-xfp*-T_ADH1_ P_PGK1_-*Ec-ackA*-T_CYC1_	This study
JMC_P4	2μ plasmid, TRP1 marker, P_GPD1_-*Bp-xfp*-T_ADH1_ P_PGK1_-*Ec-ackA*-T_CYC1_	This study
JMC_P21	2μ plasmid, URA3 marker, P_GPD1_-*ALD6*-HA-T_ADH1_	This study
JMC_P22	2μ plasmid, URA3 marker, P_GPD1_-*Bp-xfp*-T_ADH1_ P_PGK1_-*Bs-pta-*T_CYC1_	This study
JMC_P26	2μ plasmid, URA3 marker, P_TPI1_-*CAB1*-T_PGK1_	This study
JMC_P28	2μ plasmid, URA3 marker, P_GPD1_-*Bp-xfp*-T_ADH1_ P_PGK1_-*Ec-ackA*-T_CYC1_ P_TPI1_-*CAB1*-T_PGK1_	This study
JMC_P39	2μ plasmid, URA3 marker, P_GPD1_-*Bp-xfp*-T_ADH1_ P_PGK1_-*Bs-pta*-T_CYC1_ P_TPI1_-*CAB1*-T_PGK1_	This study
Sip_L33	2μ plasmid, URA3 marker, P_GPD1_-*ERG1*–T_ADH1_ P_TEF1_-*ERG13*-T_ACT1_ (P_TEF1_-*HMG1*-T_CYC1_)	This study
p426ADH	2μ plasmid, URA3 marker, P_ADH_-empty -T_CYC1_	(Mumberg et al., 1995)
p39	2μ plasmid, URA3 marker, P_GPD1_-empty-T_ADH1_	This study
pYZ23	Plasmid containing Lox71-bleMX6-Lox66 δ-integration cassette	(Zhao et al., 2018)
JMC_P10	Plasmid for δ-integration of P_GPD1_*-Ef-mvaE*-T_ADH1_ P_TEF1_-Ef-*mvaS*-T_ACT1_ P_PGK1_-*Se-acs^L641P^*-T_CYC1_	This study
pV1382_ERG9_1	CRISPR plasmid for replacement of endogenous P_ERG9_ with P_MET3_, URA3 marker	This study
CRISPR_ERG9_RT_1	CRISPR repair template for replacement of endogenous P_ERG9_ with P_MET3_, HIS3 marker	This study

Enzymes were purchased from New England Biolabs, *Inc.* (NheI, XhoI, SacI, XmaI, AscI, PmeI, T4-DNA ligase, and Phusion polymerase) or Thermo Fisher Scientific, *Inc*. (MreI), and reactions were carried out following manufacturers' instructions. Oligonucleotides used as primers were synthesized by Integrated DNA Technologies. Propagation and cloning of plasmids were carried out using the DH5α strain of *E. coli* from New England Biolabs, at 37°C in Luria-Bertani (LB) medium containing 100 μg/ml ampicillin.

Endogenous genes from the yeast PDH bypass (*ALD6*), coenzyme A biosynthetic pathway (*CAB1*), and mevalonate pathway (*ERG10, ERG13*, and *tHMG1*) were amplified with PCR from CEN.PK2-1C genomic DNA. Amplified genes were purified using gel extraction performed with the QIAquick Gel Extraction Kit from QIAGEN N.V. PCR employed primers containing NheI and XhoI restriction sites, which were used to insert genes into pJLA vectors. The primer sequences used are detailed in Table 2.

Genes from other organisms (*mvaE, mvaS, acs, xfp, ack*) were synthesized by Bio Basic *Inc.*, with codons optimized for *S. cerevisiae*. These genes were designed with flanking NheI and XhoI sites at the 5′ and 3′ ends, respectively (used to insert them into pJLA121 or pJLA123 vectors). Other heterologous genes, such as *pta* from *Bacillus subtilis*, were optimized for *S. cerevisiae* in our laboratory and purchased as gBlocks^®^ gene fragments from Integrated DNA Technologies. (Coralville, IA, USA). These gBlocks^®^ gene fragments were synthesized with 40 bp homologous flaking sequences on both *N* and *C* termini for gene insertion into pJLA121 vector through Gibson Assembly.

### Gene Deletions and δ-Integration

*ADH1* and *GPD1* were deleted by homologous recombination using KanMX (G418) and HygB (Hygromycin) resistance markers, respectively. Gene deletions were confirmed by genotyping. Strains with δ-integrations were selected in YPD agar plates supplemented with 1,600 μg/ml of Zeocin. Multiple colonies (∼12–24) were first screened to identify the highest producers, which were then used in fermentations conducted with 3 or more replicates to obtain the reported results.

### DNA Repair Template and CRISPR-Cas9 Plasmid for Genome Edition

The Cas9 and sgRNA expression vector pV1382, was kindly provided by Dr. Valmik Vyas (Vyas et al., 2015). The sgRNA sequence was designed to target the 5′ end of the ERG9 coding region (5′-CACAGCAGCAAACGATCTGGAGG-3′), and ordered as two oligomers containing 5′-phosphorylation. The two 5′-phosphorylated DNA oligoes were annealed together through incubation in a thermocycler at 37°C for 30 min, 95°C for 5 min, and then cooling to 16°C at the rate of 0.1°C/s. After digesting pV1382 with BsmBI following NEB's protocol, the annealed DNA oligoes were ligated to the digested pV1382 using T4 DNA ligase in a thermocycler at 16°C for 30 min, 65°C for 10 min, and then cooling to 25°C.

The repair template, CRISPR-*ERG9*-RT, was composed of three DNA segments—a truncated 3′ DNA segment of *PTH1* followed by P*_ERG9_, HIS3* marker cassette, and a truncated 5′ DNA segment of *ERG9* placed behind the P*_MET3_* (Supplementary Sequence 1). This construct allowed the replacement of the native promoter with pMET3-ERG9, while also introducing a mismatch in the PAM-site to prevent subsequent CRISPR-mediated DNA cutting following successful integration. The repair template was prepared separately using NEB's Phusion DNA polymerase to clone the above DNA segments from yeast genome and pYZ16 (Zhao et al., 2018), which contained the *HIS3* marker cassette. After gel purification, these three DNA segments were used to construct CRISPR-*ERG9*-RT through modified long PCR-based fusion (Shevchuk et al., 2004). The pV1382-*ERG9* CRISPR plasmid was cotransformed with this CRISPR-*ERG9*-RT *S. cerevisiae* CEN.PK2-1C and the *adh1Δ/gpd1Δ* double deletion strain (JAy498) using lithium acetate transformation. Strains were selected for their ability to grow on SC-His-Met medium, suggesting correct incorporation of the repair template. Integrations were verified using PCR amplification of the repaired region of the genome followed by DNA sequencing.

### Yeast Cultivation and Transformation

*S. cerevisiae* CEN.PK2-1C (MATa, *ura3*-52, *trp1*-289, *leu2*-3112, *his3*Δ1, MAL2-8c, SUC2) was used as the parent strain for all yeast strain constructions (all strains used in the current study are described in Table 3). Strains were streaked from glycerol stocks onto YPD or SC-dropout agar plates and incubated at 30°C for 48–72 h, or until individual colonies were clearly visible. Single colonies were used to inoculate 5–7 ml of YPD or SC-dropout medium in glass tubes, and these precultures were incubated at 30°C with 200 rpm shaking overnight (∼18 h). For five transformations, 1.5 ml preculture was added to 50 ml YPD or SC-dropout media in a 500 ml glass Erlenmeyer flask, and grown with 200 rpm shaking at 30°C until the cell optical density (OD) reached 1.0–1.5, usually 3–5 h. Strains were then transformed by standard lithium acetate procedure (Gietz & Woods, 2001; Gietz & Woods, 2002). To select for DNA transformants containing the plasmids of interest, yeast cells were plated on SC dropout medium plates. Plates were incubated for 2–3 days until sizable colonies appeared. For the strains constructed through δ-integration or gene knockout, the transformed strains were incubated in 1 ml YPD or SC-dropout media without antibiotics at 30°C for 2–3 h after the heat shock. Those strains were first plated on YPD or SC-dropout agar plates without antibiotics to grow at 30°C overnight and then replicated onto corresponding agar plates containing antibiotics for strain selection. All plates were incubated at 30°C for another 2–3 days until sizable colonies appeared.

**Table 2.** Primer Sequences

Gene	Forward	Reverse
*ALD6*	ATCATGGCTAGCAAGCTACACTTTGACACTGC	TTTTTGCTCGAGCTTAATTCTGACAGC
*CAB1*	GCTAACGCTAGCCCGCGAATTACTCAAGAGATATCT	TTCAGTCTCGAGCGTACTTGTTTTCTTAGTAGATGAATGACG
*ERG10*	AGTCTGCTAGCCAGAACGTTTACATT	AGTCTCTCGAGTATCTTTTCAATGAC
*ERG13*	AGTCTGCTAGCACTAAACTTTGTTGG	AGTCTCTCGAGTTTTTTAACATCGTA
*tHMG1*	AGTCTGCTAGCGACCAATTGGTGAAAACTG	CTCGCCTCGAGGGATTTAATGCAGGT

**Table 3.** Yeast Strains Used in This Study

Strain	Description	Genotype	Source
**CEN.PK2-1C**	Wild type	*MATa ura3-52 trp1-289 leu2-3_112 his3Δ1 MAL2-8C SUC2*	(Entian & Kötter, 2007)
**JAy498**	*adh1Δ gpd1Δ*	CEN.PK2-1C *adh1::KanMX, gpd1::HygB*	This study
**YLG6**	WT (P426ADH)	CEN.PK2-1C (P426ADH—empty vector)	This study
**SIY2**	WT (pVSLS22)	CEN.PK2-1C (*Ef-mvaE, Ef-mvaS, Se-acs^L641P^*)	This study
**SIY23**	WT (Sip_L33)	CEN.PK2-1C (*ERG10, ERG13, tHMG1*)	This study
**JCY4**	WT (pVSLS19)	CEN.PK2-1C (*Ef-mvaE, Ef-mvaS*)	This study
**JCY8**	*adh1Δgpd1Δ* (pVSLS19)	JAy498 (*Ef-mvaE, Ef-mvaS*)	This study
**JCY9**	*adh1Δgpd1Δ* (pVSLS22)	JAy498 (*Ef-mvaE, Ef-mvaS, Se-acs^L641P^*)	This study
**JCY17**	WT (JMC_P4, pVSLS22)	CEN.PK2-1C (EfMvaE, EfMvaS, SeAcsL641P) (BpXfp, EcAckA)	This study
**JCY31**	*P_MET3_-ERG9* (pVSLS22)	JCY26-1 (*Ef-mvaE, Ef-mvaS, Se-acs^L641P^*)	This study
**JCY33**	*adh1Δgpd1Δ P_MET3_-ERG9* (pVSLS22)	JCY28-1 (*Ef-mvaE, Ef-mvaS, Se-acs^L641P^*)	This study
**JCY37**	*adh1Δgpd1Δ δ-Ef-mvaE, Ef-mvaS, Se-acs^L641P^*	JAy498 *δ-Ef-mvaE, Ef-mvaS, Se-acs^L641P^::lox71-bleMX6-lox66*	This study
**JCY46**	*adh1Δgpd1Δ P_MET3_-ERG9 δ-Ef-mvaE, Ef-mvaS, Se-acs^L641P^*	JCY28-1 *δ-Ef-mvaE, Ef-mvaS, Se-acs^L641P^::lox71-bleMX6-lox66*	This study
**JCY48**	*P_MET3_-ERG9 δ Ef-mvaE, Ef-mvaS, Se-acs^L641P^*	JCY26-1 *δ-Ef-mvaE, Ef-mvaS, Se-acs^L641P^::lox71-bleMX6-lox66*	This study
**JCY51**	*δ-Ef-mvaE, Ef-mvaS, Se-acs^L641P^*	CEN.PK2-1C *δ-Ef-mvaE, Ef-mvaS, Se-acs^L641P^::lox71-bleMX6-lox66*	This study
**JCY54**	*P_MET3_-ERG9 δ-Ef-mvaE, Ef-mvaS, Se-acs^L641P^* (pVSLS32)	JCY48-A6 (*Bp-xfp, Ec-ackA*)	This study
**JCY65**	*P_MET3_-ERG9 δ-Ef-mvaE, Ef-mvaS, Se-acs^L641P^* (JMC_P21)	JCY48-A6 (*ALD6*)	This study
**JCY67**	*P_MET3_-ERG9 δ-Ef-mvaE, Ef-mvaS, Se-acs^L641P^* (JMC_P22)	JCY48-A6 (*Bp-xfp, Bs-pta*)	This study
**JCY73**	*P_MET3_-ERG9 δ-Ef-mvaE, Ef-mvaS, Se-acs^L641P^* (p39)	JCY48-A6 (empty vector)	This study
**JCY87**	*P_MET3_-ERG9 δ-Ef-mvaE, Ef-mvaS, Se-acs^L641P^* (JMC_P26)	JCY48-A6 (*CAB1*)	This study
**JCY88**	*P_MET3_-ERG9 δ-Ef-mvaE, Ef-mvaS, Se-acs^L641P^* (JMC_P28)	JCY48-A6 (*Bp-xfp, Ec-ackA, CAB1*)	This study
**JCY90**	*adh1Δgpd1Δ δ-Ef-mvaE, Ef-mvaS, Se-acs^L641P^* (p39)	JCY37-D3 (empty vector)	This study
**JCY91**	*adh1Δgpd1Δ δ-Ef-mvaE, Ef-mvaS, Se-acs^L641P^* (pVSLS32)	JCY37-D3 (*Bp-xfp, Ec-ackA*)	This study
**JCY93**	*δ-Ef-mvaE, Ef-mvaS, Se-acs^L641P^* (p39)	JCY51-A6 (empty vector)	This study
**JCY94**	*δ-Ef-mvaE, Ef-mvaS, Se-acs^L641P^* (pVSLS32)	JCY51-A6 (*Bp-xfp, Ec-ackA*)	This study
**JCY98**	*δ-Ef-mvaE, Ef-mvaS, Se-acs^L641P^* (pVSLS22)	JCY51-A6 (*Ef-mvaE, Ef-mvaS, Se-acs^L641P^*)	This study
**JCY99**	*adh1Δgpd1Δ δ-Ef-mvaE, Ef-mvaS, Se-acs^L641P^* (pVSLS22)	JCY37-D3 (*Ef-mvaE, Ef-mvaS, Se-acs^L641P^*)	This study
**JCY110**	*P_MET3_-ERG9 δ-Ef-mvaE, Ef-mvaS, Se-acs^L641P^* (JMC_P39)	JCY48-A6 (*Bp-xfp, Bs-pta, CAB1*)	This study
**JCY112**	*P_MET3_-ERG9 δ-Ef-mvaE, Ef-mvaS, Se-acs^L641P^* (pVSLS22)	JCY48-A6 (*Ef-mvaE, Ef-mvaS, Se-acs^L641P^*)	This study
**JCY113**	*adh1Δgpd1Δ δ-Ef-mvaE, Ef-mvaS, Se-acs^L641P^* (JMC_P22)	JCY37-D3 (*Bp-xfp, Bs-pta*)	This study
**JCY115**	*adh1Δgpd1Δ δ-Ef-mvaE, Ef-mvaS, Se-acs^L641P^* (JMC_P21)	JCY37-D3 (*ALD6*)	This study
**JCY116**	*adh1Δgpd1Δ δ-Ef-mvaE, Ef-mvaS, Se-acs^L641P^* (JMC_P26)	JCY37-D3 (*CAB1*)	This study
**JCY117**	*δ-Ef-mvaE, Ef-mvaS, Se-acs^L641P^* (JMC_P22)	JCY51-A6 (*Bp-xfp, Bs-pta*)	This study
**JCY119**	*δ-Ef-mvaE, Ef-mvaS, Se-acs^L641P^* (JMC_P21)	JCY51-A6 (*ALD6*)	This study
**JCY120**	*δ-Ef-mvaE, Ef-mvaS, Se-acs^L641P^* (JMC_P26)	JCY51-A6 (*CAB1*)	This study

### High Cell-Density Fermentations: Strain Selection

After yeast cells were transformed with the appropriate plasmid, 12–24 different colonies were picked randomly and used to inoculate 1 ml of SC-complete or SC-dropout liquid media in 24-well cell culture plates for overnight culture (16–20 hrs) at 30°C. The next day, 5 μL and 20 μL of the overnight culture was transferred to agar plates and new 24-well cell culture plates for stock and fermentation, respectively. The cultures in the 24-well plates were first incubated at 30°C for 1 or 2 days. After centrifuging the cells and removing old liquid media, the same volume fresh media was added to resuspend the cells for 2-day high cell-density fermentations at 30°C and shaking at 250 RPM. At the end of the fermentation, mevalonate was quantified as described below. The strains which produced mevalonate at the highest titers were sampled from the agar plates and stored in 30% glycerol at −80°C.

### Low Cell-Density 7-Day Fermentation

Following strain selection, different strains were compared for their ability to produce mevalonate through the course of a 7-day fermentation. First, we inoculated strains from glycerol stock onto YPD or SC-dropout agar plates for colony formation. After sizable colonies were formed, three different colonies of each strain were inoculated into 24-well culture plates which contained 1 ml SC-dropout media with 2% glucose. The plates were incubated with shaking at 250 RPM at 30°C overnight. These seeds were then used to inoculate 10 ml of SC-dropout media with 2% glucose in 50 ml Falcon tubes to an initial OD_600_ of 0.1 and incubated with shaking at 250 RPM and 30°C. The cultures were loosely capped during the course of fermentation to allow for semi-aerobic conditions. Mevalonate production was assessed at the end of the 7-day fermentation using the methods described below.

### Fed-Batch Fermentation in Bioreactor

For fed-batch fermentations, seed cultures were prepared by streaking 5 μL of frozen cells in 30% glycerol (vol/vol) onto an appropriate SC-dropout agar plate. After 2-day incubation at 30°C, seed colonies were used to inoculate 5 ml liquid SC-dropout media with 2% glucose and cultured for 10 h. They were subcultured again by transferring 1 ml of the 10-h culture into 50 ml liquid SC-dropout media with 2% glucose and grown overnight. The next day, the overnight culture was used to inoculate the 2 L fermentor (Eppendorf BioFlo^®^ 120, Hamburg, Germany) containing 1 L fermentation media (SC-dropout media containing 20 g/l glucose, 15 μM pantothenate, and 2 mM methionine). Fermentation was carried out at 30°C with an agitation speed of 200 rpm, gas flow rate of 3.0 standard liters per minute. with 100% air, and OD_600_ value starting at 0.5. The pH was maintained at pH 5.0 by automatic addition of 1 M KOH, while the dissolved oxygen (DO) value was kept at 25% by automatic adjustment of agitation speed. Feeding fresh media, which was twofold concentrated SC-dropout media containing 400 g/l glucose, 15 μM pantothenate, and 50 mM methionine, began when glucose in the original SC-dropout media was exhausted, usually between the 12th and 16th h of the fermentation. The feed flow rate was 4.5 ml/h to deliver a 1.8 g/h of glucose. The mevalonate concentration in the culture broth was determined by HPLC and measured in triplicate. For these experiments, three identical bioreactor runs were undertaken and averaged.

### Analytical Methods

Concentrations of mevalonate, ethanol, glycerol, and glucose were measured with HPLC. At the end of fermentation, 1 ml cultures were centrifuged at 12,500 g for 30 min, and the supernatant taken for HPLC analysis. HPLC was conducted using an Agilent 6100 system equipped with a BioRad Aminex HPX-87H column (300 × 7.8 mm, 9 μm particle size, 0.25 mm diameter) and a refractive index detector (RID). The mobile phase was 5 mM H_2_SO_4_ in deionized water, with flow rate 0.6 ml/min. The column and detector temperatures were set at 55°C. The peak areas of each compound were compared against calibration curves prepared with commercial standards. Mevalonate was quantified by use of mevalonolactone equivalents—mevalonolactone being the lactone version of mevalonic acid (mevalonate), which is produced when mevalonic acid is exposed to acidic conditions. As yeast culture is naturally acidic, we assumed that the majority of secreted mevalonate was converted to this form. To quantify cell densities, we measured the optical density (OD_600_) of cell cultures, compared to a blank media sample, using an Eppendorf BioSpectrometer. Statistics were computed via student’s t-test from biological triplicates unless otherwise specified.

## Results

### Comparison of the Mevalonate Pathways from *E. faecalis* and *S. cerevisiae*

**Fig. 2:**
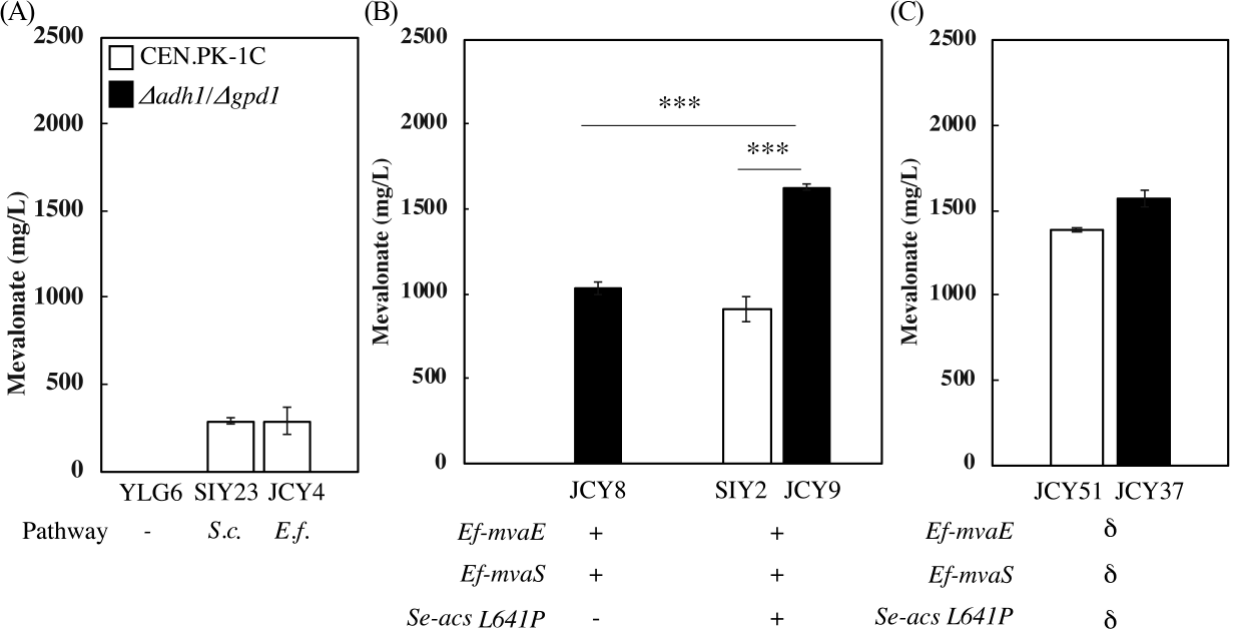
Establishment of a minimal cassette for high-level mevalonate production. (A) Comparison between the mevalonate pathways from *Saccharomyces cerevisiae* and *Enterococcus faecalis*. YLG6 is a negative control containing an empty 2μ plasmid. The average mevalonate production of six independent colonies, following a 2-day high cell-density fermentation. Error bars represent one standard deviation. (B) The *E. faecalis* mevalonate pathway expressed alone or with the inhibition-insensitive *Se-acs*^L641P^ in 2μ plasmids, in wild-type or *Δadh1/Δgpd1* deletion backgrounds. (C) Strains with mevalonate cassette integrated in δ-sites. B and C show the average mevalonate production of 7-day fermentations starting from low cell density. Error bars represent one standard deviation from three biologically independent replicates. ***P < 0.001.

Many isoprenoids have been produced in *S. cerevisiae* by overexpressing a deregulated endogenous mevalonate pathway or its heterologous counterpart from *E. faecalis* but these pathways have not been directly compared for the biosynthesis of mevalonate itself. The endogenous pathway is comprised by enzymes encoded by *ERG10, ERG13*, and a truncated feedback-insensitive form of *HMG1*, to boost isoprenoid production (Polakowski et al., 1998; Ro et al., 2006). The heterologous pathway from *E. faecalis* is comprised of a bifunctional acetoacetyl-CoA thiolase and HMG-CoA reductase (Hedl et al., 2002, *Ef-mvaE*) and an HMG-CoA synthase (*Ef-mvaS*, Fig. 1). To compare the efficacy of these two pathways for mevalonate production, each of them was expressed using a high-copy 2μ plasmid in CEN.PK2-1C, a yeast strain shown to support high flux through the isoprenoid pathway (Westfall et al., 2012). We observed no difference between overexpressing the endogenous or the *E. faecalis* pathways (JCY4 vs. SIY23), with either of them producing approximately 10-fold higher titers (Fig. 2A) than previously reported (Rodriguez et al., 2016). Mevalonate production in the wild-type strain transformed with an empty plasmid (YLG6) was undetectable. As the heterologous pathway from *E. faecalis* is less likely to be subject to endogenous regulation, we used the *Ef-mvaE/Ef-mvaS* enzymes for further strain development. This established a suitable mevalonate platform from which we could manipulate acetyl-CoA supply to further increase production levels.

### Disrupting Overflow Metabolism and Establishing a Minimal Cassette for Mevalonate Production

A major challenge in *S. cerevisiae* metabolic engineering is the strong drive in this yeast to convert fermentable sugars to overflow metabolic products (i.e., ethanol and glycerol). We sought to reduce the formation of these byproducts by deleting the major isoforms of alcohol and glycerol-3-phosphate dehydrogenases (*ADH1* and *GPD1*, Albertyn et al., 1994; de Smidt et al., 2012), as reducing their activity has been shown to increase acetyl-CoA synthesis (Lian et al., 2014). As expected, overexpressing the *E. faecalis* mevalonate pathway in CEN.PK2-1C in a *Δadh1/Δgpd1* double deletion strain (JCY8) increases mevalonate titers to 1,030 ± 30 mg/l in a 7-day fermentation (Fig. 2B, Supplementary Fig. S1). To better compete with overflow metabolism, we introduced a feedback-inhibition resistant *acs* mutant from *S. enterica* (*Se-acs^L641P^*) (Starai et al., 2005). Indeed, overexpressing *Se-acs^L641P^* in CEN.PK-1C (SIY2) increases mevalonate production to 910 ± 70 mg/l, while overexpressing it in the *Δadh1/Δgpd1* deletion strain (JCY9) further improves it to 1,620 ± 20 mg/l, (Fig. 2B, p-value < 0.001 for both JCY9 vs. SIY2, and JCY9 vs. JCY8). This corresponds to mevalonate yields of 0.05 ± 0.01 and 0.08 ± 0.01 g-mevalonate/g-glucose, or 8.3% and 14.8% of the theoretical maximum yield for the wild-type (SIY2) and *Δadh1/Δgpd1* deletion (JCY9) strains, respectively. The *Δadh1/Δgpd1* strain shows a significant growth defect and delay in glucose consumption and, while its reduced ethanol production boosts mevalonate titers, its glycerol production is also increased, likely due to *GPD2* (Supplementary Fig. S1). This shows that there is an additive effect between reducing overflow metabolism toward ethanol production and increasing the pull toward acetyl-CoA in enhancing mevalonate production.

In an effort to stabilize multiple copies of the mevalonate pathway in the yeast genome, we integrated *Ef-mvaE, Ef-mvaS*, and *Se-acs^L641P^* into δ-sites (YARCdelta5). This method, called δ-integration, targets the long terminal repeats (LTRs) of Ty1 transposable elements distributed across the genome to introduce multiple copies of exogenous sequences. δ-Integration has been used to enhance production of various chemicals in yeast, including isoprenoids (Shao et al., 2009; Zhao et al., 2018). As expected, δ-integration of the mevalonate cassette (*Ef-mvaE, Ef-mvaS*, and *Se-acs^L641P^*) increases mevalonate production in the *Δadh1/Δgpd1* strain JCY37 to levels comparable to those obtained with 2μ plasmids (1,570 ± 50 mg/l), but without the need for constant media selection. Interestingly, δ-integration in CEN.PK-1C results in a strain (JCY51) that produces significantly more mevalonate (1,390 ± 10 mg/l, p-value < 0.001) than the strain (SIY2) transformed with the pathway in a 2μ plasmid (910 ± 70 mg/l, Fig. 2C). We hypothesize that the higher production observed in strains transformed by δ-integration could be due to a combination of factors, including better strain stability, a more favorable copy-number of mevalonate pathway genes, and lower genetic burden. We exploited this stable integration of the mevalonate cassette in δ-sites to further develop yeast strains with enhanced mevalonate production.

### Engineering a Bifid Shunt, PDH Bypass, and CoA Synthesis in Mevalonate Production Strains

**Fig. 3:**
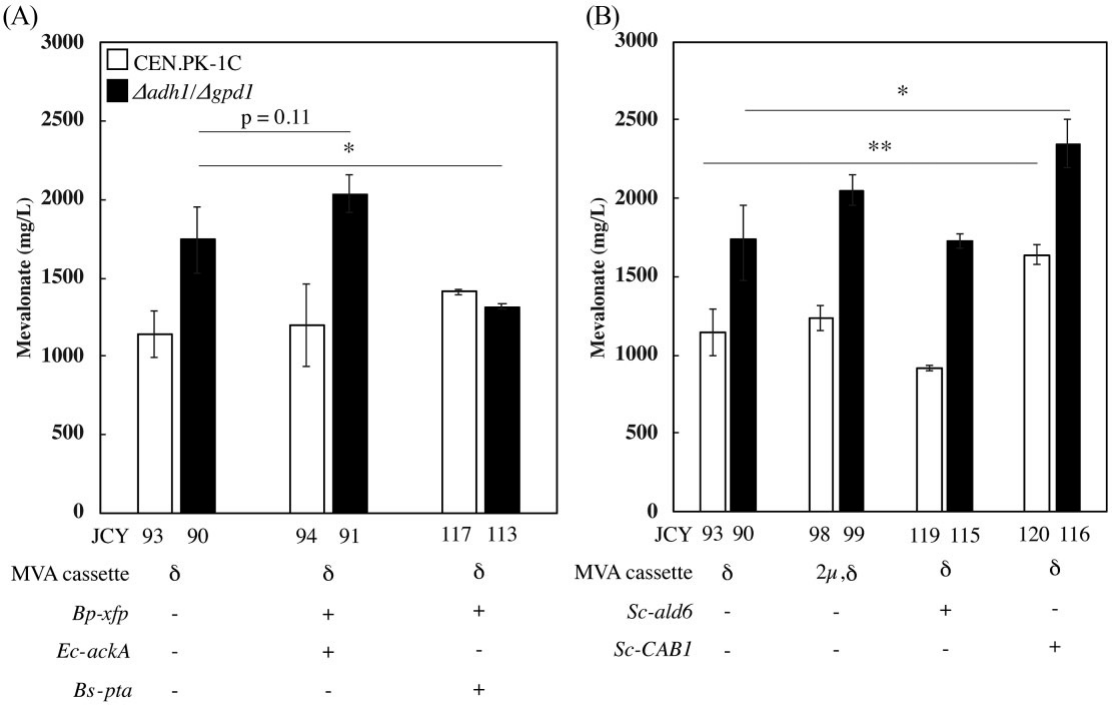
Introduction of pathways to increase acetyl-CoA synthesis in mevalonate-producing strains using 2μ plasmids. (A) Introduction of bifid shunt pathway enzymes. (B) Overexpression of *ALD6* aiming to enhance the PDH-bypass or *CAB1* to boost CoA synthesis. The mevalonate cassette (*Ef-mvaE, Ef-mvaF, Sc-acs^L641P^*) is integrated in d-sites in all strains; JCY98 and JCY99 also contains it in a 2μ plasmid. Fermentations were carried out for 7 days starting from low cell density. Error bars represent one standard deviation from three biologically independent replicates. *P < 0.05, **P < 0.01.

We next tested whether additional improvements to acetyl-CoA supply could be achieved by introducing a heterologous bifid shunt or further engineering of the PDH bypass. Expression of the xylulose-5-phosphate phosphoketolase (*Bp-xfp*) from *Bifidobacterium pseudolongum* and acetate kinase (*Ec-ackA*) from *E. coli* did not significantly increase mevalonate production (Fig. 3A). Replacing *Ec-ackA* with expression of the phosphotransacetylase (*Bs-pta*) from *B. subtilis* also failed to improve mevalonate production in the wild-type background, and actually reduces titers by 25% (1,320 ± 20 mg/l) in the *Δadh1/Δgpd1* background (p-value < 0.05 between JCY113 and strain transformed with empty vector, JCY90). This result was unexpected, as these enzymes, including the same *Bs-pta*, have been reported to increase the production of acetyl-CoA-derived chemicals in *S. cerevisiae* (de Jong et al., 2014; Sonderegger et al., 2004). Furthermore, expressing *Bp-xfp* and *Ec-ackA* using a 2μ plasmid in strains expressing the mevalonate cassette in a separate plasmid enhances mevalonate production, which confirms that these enzymes are active in *S. cerevisiae* (Supplementary Fig. S2). However, redirection of metabolism through the bifid shunt was not effective at increasing mevalonate titers in higher-producing strains, suggesting that there is a shift in the metabolic bottleneck.

Our efforts to further enhance the endogenous PDH bypass gave additional evidence to support this hypothesis. Introduction of additional copies of the mevalonate cassette in a 2μ plasmid with (JCY 98) or without *ADH1* and *GPD1* (JCY99) also failed to improve mevalonate production (Fig. 3B). The small increase in production in the *Δadh1/Δgpd1* strain (2,050 ± 100 mg/l) is not statistically significant (p-value = 0.09). Additionally, overexpression of the major aldehyde dehydrogenase isoform (*ALD6*) does not increase mevalonate production (Fig. 3B), which is also unexpected based on previous reports that overexpression of *Se-acs^L641P^* and *ALD6* improves production of amorphadiene and alpha-santalene (Chen et al., 2013; Shiba et al., 2007). These results are consistent with a new metabolic bottleneck in these high-producing strains.

A possible bottleneck in these strains is CoA availability. To test this hypothesis, we overexpressed pantothenate kinase (*CAB1*), which revealed that indeed enhancing CoA biosynthesis increases mevalonate production in these strains. This is true for both the wild-type and *Δadh1/Δgpd1* backgrounds, yielding 1,640 ± 70 mg/l (JCY93 vs. JCY120, p-value < 0.01) and 2,350 ± 160 mg/l, respectively (Fig. 3B, JCY90 vs. JCY116, p-value < 0.05). These results confirm that CoA is limiting in our strains, and that boosting its biosynthesis increases flux toward acetyl-CoA with significant effects in mevalonate production.

### Inhibition of *ERG9* Improves Mevalonate Production

**Fig. 4:**
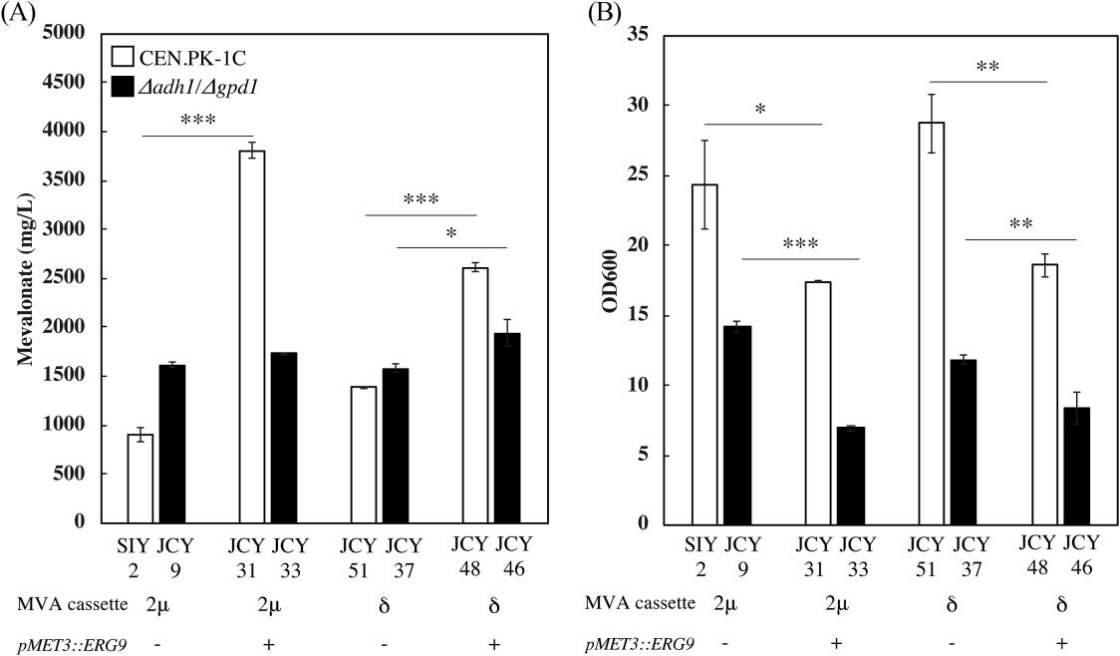
Effect of *ERG9* inhibition on mevalonate production. (A) Mevalonate production with the mevalonate cassette introduced through either 2μ plasmid or δ-integration. To repress *ERG9*, 2 mM methionine was added at the beginning of the 7-day fermentation (see methods). (B) Final cell densities accumulated at the end of 7-day fermentations, measured immediately prior to sampling for mevalonate production. Error bars represent one standard deviation from the average of three biologically independent replicates. ***P < 0.001.

We next tested whether manipulating the pathway downstream of mevalonate improves production. Repression of squalene synthase (*ERG9*) has been shown to increase production of mevalonate, sesquiterpenes, and diterpenes in *S. cerevisiae* (Callari et al., 2018; Jakočiūnas et al., 2015; Paradise et al., 2008; Ro et al., 2006). Using the methionine-repressible P_MET3_ to express *ERG9* allowed us to repress its transcription by adding 2 mM methionine to the media. As expected, inhibition of *ERG9* significantly improves mevalonate production in wild-type strains carrying the mevalonate cassette in 2μ plasmids or δ-sites, reaching as much as 3,810 ± 70 and 2,610 ± 40 mg/l, respectively (Fig. 4A). This corresponds to as much as a 4.2-fold increase (in strain using 2μ plasmid) over the nonrepressed strains. However, overexpression of bifid shunt enzymes (*Bp-xfp* and *Ec-ackA*, or *Bs-pta*), *ALD6* to enhance the PDH bypass, or *CAB1* to boost CoA biosynthesis did not further increase mevalonate production (Supplementary Fig. S3). Surprisingly, the effect of repressing *ERG9* in the *Δadh1/Δgpd1* strain was minimal, with only a small increase in mevalonate production observed in strains carrying the mevalonate cassette in δ-sites (p-value < 0.05). The final biomass accumulated was significantly decreased in strains carrying the *Δadh1/Δgpd1* double deletion with *ERG9* repression (Fig. 4B). It is unclear why there is such a vast difference in the effect of *ERG9* repression between these strains, but this exacerbated growth defect in the *Δadh1/Δgpd1* strains offers a likely explanation.

### Improving Mevalonate Production Through Medium Supplementation and Carbon Source

**Fig. 5:**
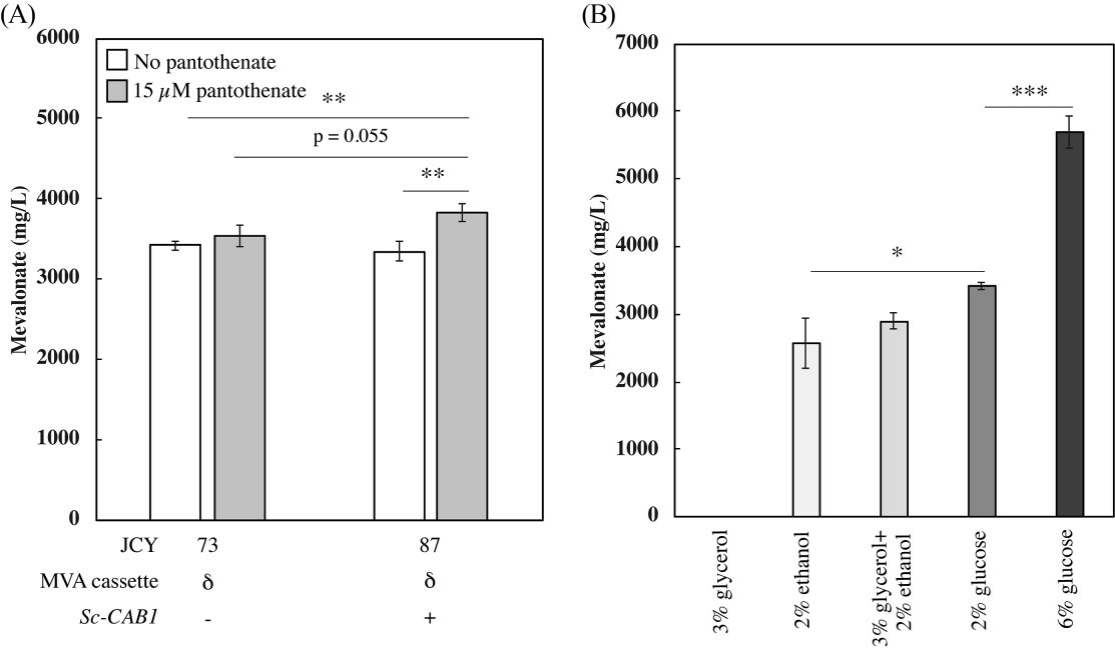
Exploring mevalonate production with pantothenate supplementation and different carbon sources in the context of *ERG9* repression. (A) Mevalonate titers with 2 mM methionine added at the beginning of fermentation in cell lines expressing either an empty plasmid (JCY73) or *CAB1* (JCY87) and with or without 15 μM pantothenate. (B) Mevalonate production in JCY73 fermentations using SC-Ura medium with different carbon sources. All fermentations were carried out for 7 days starting from low cell density. Fermentations containing 3% glycerol as the sole carbon source did not result in detectable levels of mevalonate production. Error bars represent one standard deviation from the average of three biologically independent replicates. **P < 0.01, ***P < 0.001.

Considering the possibility that the effect of *CAB1* overexpression on mevalonate production was limited by its substrate availability, we next explored adding pantothenate to the fermentation media. We found that *CAB1* overexpression combined with pantothenate supplementation is sufficient to increase mevalonate production to 3,830 ± 120 mg/l (Fig. 5A), which is a 12% increase relative to controls without either intervention (JCY73 no pantothenate vs. JCY87 with pantothenate, p-value < 0.01) and 15% increase relative to the strain overexpressing *CAB1* (JCY87) but without pantothenate supplementation (p-value < 0.01). This corresponds to mevalonate yields of 0.19 ± 0.01 g-mevalonate/g-glucose, which is 35% of the theoretical maximum. Additionally, this effect requires *CAB1* overexpression, as the control strain (JCY73) does not show a similar increase upon exposure to pantothenate. Unfortunately, even in this context, the bifid shunt enzymes failed to improve mevalonate titers (Supplementary Fig. S4). Nevertheless, these results demonstrate that pantothenate supplementation combined with *CAB1* overexpression can be an effective strategy to further increase mevalonate production.

Finally, we explored how different carbon sources affect mevalonate production. We found that while glycerol alone is a poor carbon source for mevalonate production (not detectable by HPLC), ethanol is an effective feed source. Yeast cultures using 2% ethanol as carbon source with or without 3% glycerol supplementation produced 2,900 ± 130 and 2,570 ± 370 mg/l of mevalonate, respectively. However, fermentations using 2% glucose produced 33% higher mevalonate compared to 2% ethanol, resulting in 3,420 ± 60 mg/l (p < 0.05, Fig. 5B). We next tested whether increasing glucose concentration would further increase mevalonate production. Using 6% glucose results in a 1.7-fold increase in mevalonate titers to 5,700 ± 240 mg/l (p-value < 0.001). However, the higher glucose concentration leads to a reduction in mevalonate yields from 0.17 ± 0.01 g-mevalonate/g-glucose (in 2% glucose) to 0.10 ± 0.01 g-mevalonate/g-glucose, which is 31.2% and 17.3% of the theoretical maximum, respectively. These results suggest that glucose is indeed the preferred feedstock for mevalonate production and that increasing glucose concentrations increases titers, but at the expense of yields.

### Mevalonate Production in Fed-Batch Bioreactor

**Fig. 6:**
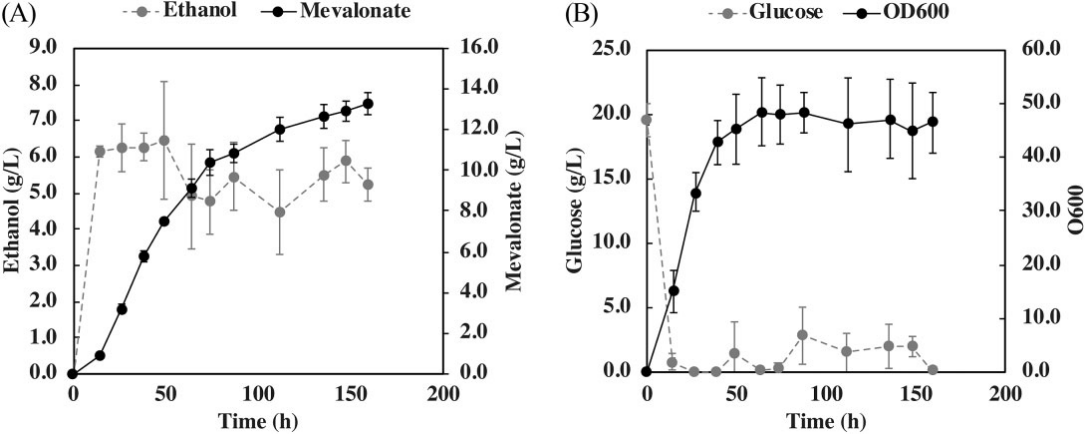
Mevalonate production using strain JCY87 in 2 L fed-batch bioreactors operated initially in 20 g/l glucose, 15 μM pantothenate, and 2 mM methionine. At 14 h, a 4.5 ml/h feed was started containing 40% glucose in twofold concentrated SC-Ura media containing 15 μM pantothenate and 50 mM methionine. (A) Mevalonate and ethanol production. (B) Glucose concentration and cell density in the bioreactor. The average values of three independent bioreactor experiments, with error bars representing the standard deviation. See methods for complete details.

To maximize mevalonate production, we carried out fermentations in a fed-batch bioreactor. In these experiments, we used strain JCY87, which has the mevalonate cassette integrated in δ-sites, a 2μ plasmid to overexpress *CAB1*, and the P_MET3_ promoter controlling the endogenous copy of *ERG9.* Using a constant feed of 1.8 g/h glucose (in twofold concentrated SC-Ura with 40% glucose, methionine at 50 mM, and pantothenate at 15 μM) in a 2 L bioreactor results in the production of 13.3 ± 0.50 g/l of mevalonate in 160-h fermentations (Fig. 6), achieving 7.5 ± 0.21 g/l in the first ∼48 h of fermentation. These results constitute the highest mevalonate titers, yields, and productivities reported in *S. cerevisiae* thus far, demonstrating the feasibility of using this yeast for high-level mevalonate production.

## Discussion

Here we show that *S. cerevisiae* can be engineered to produce relatively high levels of mevalonate. To our knowledge, only two studies have focused on producing mevalonate as an end product in *S. cerevisiae*: the first study used *IDH1* deletion, *Ef-mvaE/S* and *ACL* overexpression to achieve ∼35 mg/l production (Rodriguez et al., 2016), while the other utilized CRISPR-mediated quintuple gene knockout of *Δerg9/Δyjl064w/Δrox1/Δypl062w/Δbts1* to improve production to ∼2 mg/l without overexpressing any genes in the mevalonate pathway (Jakočiūnas et al., 2015). Two additional studies analyzed mevalonate production as an intermediate metabolite of isoprenoid biosynthesis in *S. cerevisiae*, with the highest titers being 378 mg/l during amorphadiene production (Shiba et al., 2007; Özaydın et al., 2012). Here we show that combining the heterologous expression of the *E. faecalis* mevalonate pathway, with a partial PDH bypass, enhancement of CoA biosynthesis, and *ERG9* repression results in the production of as much as 13.3 ± 0.50 g/l of mevalonate. This constitutes an approximate 360-fold improvement on the highest titers of mevalonate as end product previously reported, which establishes *S. cerevisiae* as a viable host for high level production of mevalonate.

Only a partial PDH bypass improves mevalonate production in our strains. Overexpression of the *Se-acs*^L641P^ results in as much as a 1.5-fold increase in mevalonate production in the *Δadh1/Δgpd1* background strain. However, in our hands, overexpression of *ALD6* did not further improve, or actually hurt, production. These results differ from those in previous studies reporting that *ALD6* overexpression combined with *Se-acs^L641P^* improves amorphadiene, n-butanol, or alpha-santalene (Chen et al., 2013; Shiba et al., 2007; Krivoruchko et al., 2013). They are, however, consistent with other studies showing that *ALD6* overexpression combined with *Se-acs^L641P^* produces less n-butanol than *Se-acs^L641P^* alone (Lian et al., 2014). Nevertheless, our study reaffirms the positive impact of the PDH bypass, at least in the overexpression *Se-acs^L641P^*, to improve acetyl-CoA-derived products, in this case mevalonate.

Repression of *ERG9* also significantly enhances mevalonate production. Several studies have used this approach to improve sesquiterpene and diterpene synthesis (Callari et al., 2018; Dong et al., 2020; Paradise et al., 2008; Ro et al., 2006). We found that, after introducing the mevalonate cassette (*Ef-mvaE, Ef-mvaS, and Se-acs^L641P^), ERG9* repression makes the single most important contribution in improving mevalonate production. Two previous studies have also demonstrated that *ERG9* repression increased mevalonate production, but report maximum titers of only ∼2–30 mg/l (Özaydın et al., 2013; Jakočiūnas et al., 2015). It has been proposed that lowering Erg9p levels reduces competition for farnesyl pyrophosphate, which is the substrate for squalene synthesis (by Erg9p) as well as sesquiterpene and diterpene production (Asadollahi et al., 2010; Paradise et al., 2008). However, because mevalonate synthesis lies several steps upstream of squalene synthesis and is therefore not in direct competition with Erg9p, our results suggest that *ERG9* repression leads to the upregulation of the mevalonate pathway, probably as a response to ergosterol starvation. This is more consistent with findings that azoles, which inhibit lanosterol 14α-demethylase in ergosterol biosynthesis, upregulates sterol biosynthesis, including the upstream mevalonate biosynthesis (Dimster-Denk et al., 1999). Although the exact mechanism by which this occurs is unclear, *ERG9* repression in combination with the mevalonate cassette is highly effective at increasing production.

Consistent with acetyl-CoA availability being limiting, enhancing CoA biosynthesis also improves mevalonate production. *CAB1* overexpression and pantothenate supplementation has been previously used to improve naringenin production, although maximum titers remained relatively modest, 12.5 mg/l (Liu et al., 2017). Another study found that pantothenate supplementation improves n-butanol production, but overexpression of pantothenate kinase and polyamine oxidase achieves a titer of 243 mg/l regardless of pantothenate supplementation (Schadeweg and Boles, 2006). Here, we found that the effect of promoting CoA biosynthesis varies under different metabolic flux conditions. Overexpression of *CAB1* in a strain containing only the mevalonate cassette is enough to significantly improve mevalonate production by 44% to 1,640 ± 70 mg/l (Fig. 3B). However, overexpressing it in strains in which *ERG9* is also repressed only increases mevalonate production if pantothenate is also supplemented in the media (Fig. 5A); and even then, the improvement is more modest (only 12%). These results suggest that CoA biosynthesis is limiting in mevalonate-producing strains, whether or not *ERG9* is repressed.

An unexpected result in this study is the ineffectiveness of the bifid shunt pathway at increasing mevalonate titers. Previous studies have used this pathway to increase titers of acetyl-CoA-derived products in *S. cerevisiae* (de Jong et al., 2014; Kocharin et al., 2013; Su et al., 2020). However, in our hands, *Bp-xfp* and *Ec-ackA* increase mevalonate production only in wild-type strains containing the mevalonate cassette (Supplementary Fig. S2), but this effect is lost when overexpressing these enzymes in combination with *ADH1/GPD1* deletions (Fig. 3A), *ERG9* repression (Supplementary Fig. S3), or *CAB1* overexpression (including with pantothenate addition, Supplementary Fig. S4). This suggests that acetate levels become less limiting for acetyl-CoA synthesis in strains with these additional interventions. Expression of *Bs-pta* is also ineffective at improving mevalonate production and was even counterproductive in strains with *ADH1/GPD1* deletions or *ERG9* repression, possibly due to the enhanced flux toward acetyl-CoA in these strains. One possible explanation for this observation is the potential for *Bs-pta* to catalyze the reverse reaction at increased levels of acetyl-CoA to make acetyl-phosphate (Campos-Bermudez et al., 2010). This could also result in an ATP-consuming futile cycle in which endogenous Gpp1p and Gpp2p convert the resulting acetate phosphate to acetate, which is then converted back to acetyl-CoA by *Se-acs*^L641P^ (Bergman et al., 2016; Meadows et al., 2016). These results suggest that once the levels of acetate or acetyl-CoA are increased by other means, the activity of bifid shunt enzymes to promote acetyl-CoA-derived products can be limited or even counterproductive, at least in some strains.

Despite the significant improvement in mevalonate production we obtained compared to previous studies in S*. cerevisiae*, there is still much room for improvement. Efforts in *E. coli* have reported mevalonate titers between 30–84 g/l (Tabata & Hashimoto, 2004; Wang et al., 2016). The difficulty in achieving similar titers in *S. cerevisiae* stems largely from the strong overflow metabolism in this yeast, which diverts much of the carbon flux toward ethanol and glycerol production. While deleting *ADH1* and *GPD1* significantly improves mevalonate production, ethanol and glycerol remain major byproducts in *Δadh1/Δgpd1* strains due to several endogenous isoenzymes. Deleting additional alcohol dehydrogenases and *GPD2* could help further improve mevalonate production, but only to some extent, as the redox cofactors (NAD(P)^+^/NAD(P)H) are imbalanced in the pathway; though strategies exist to improve this balance (Bae et al., 2016; Shi et al., 2016). Another aspect limiting mevalonate production in ours strains is that acetyl-CoA synthetase (*ACS*) hydrolyzes three molecules of ATP to generate the three molecules of acetyl-CoA required for a single molecule of mevalonate, which makes the PDH bypass energetically costly (Starai and Escelante-Semererna 2004). One possible approach to address this limitation is using acetylating aldehyde dehydrogenases, which produce acetyl-CoA from acetaldehyde without expending ATP (Kozak et al., 2016). This strategy has been applied to improve production of farnesene (Meadows et al., 2016); however, caution must be exercised as another study utilized the reverse reaction of this enzyme to produce acetaldehyde from acetyl-coA derived from xylitol degradation (Sonderegger et al., 2004). Another ATP-neutral route to acetyl-CoA synthesis could be achieved with a cytosolic PDH. Indeed, this strategy has been implemented in *S. cerevisiae*, in which the PDH complex from *E. faecalis* replaced the PDH-bypass as the source of cytosolic acetyl-coA; however, this required supplementation of lipoate and overexpression of genes involved in lipoylation (Kozak et al., 2014). Therefore, strategies to increase carbon flow toward the mevalonate pathway and reduce the energy requirements of acetyl-CoA synthesis may further increase mevalonate production.

## Conclusion

We have developed a strain of *S. cerevisiae* that achieves high-level production of mevalonate, as much as 13.3 ± 0.50 g/l in fed batch lab-scale bioreactors. This strain has a mevalonate pathway, which includes the feedback-insensitive *Se-acs*^L641P^, *Ef-mvaE*, and *Ef-mvaS* genomically integrated in δ-sites. Additionally, carbon flux through the pathway in this strain is enhanced by repression of *ERG9*. Finally, CoA biosynthesis is enhanced in this strain by overexpression of *CAB1* and pantothenate supplementation. However, bifid shunt enzymes are unable to further improve mevalonate production in this strain. These interventions improve titers in *S. cerevisiae* by approximately 360-fold relative to previous reports, establishing this yeast as a viable host for mevalonate production.

## Supplementary Material

kuab050_Supplemental_File

## Data Availability

The data supporting the findings of this study are available within the paper (and its supplementary information file), but original data that supports the findings are available upon reasonable request.
