# Supplementary material for: Engineering acetyl-CoA supply and ERG9 repression to enhance mevalonate production in Saccharomyces cerevisiae
Source: J Ind Microbiol Biotechnol. 2021 Aug 5;48(9-10):kuab050. doi: 10.1093/jimb/kuab050 (PMC8788843; doi:10.1093/jimb/kuab050)
Supplement: kuab050_Supplemental_File [file kuab050_supplemental_file.docx]

**­Supplementary Information:**

**Engineering acetyl-CoA supply and *ERG9* repression to enhance mevalonate production in *Saccharomyces cerevisiae*.**

Scott A. Wegner^1†^, Jhong-Min Chen^2†^, Samantha S. Ip^2^, Yanfei Zhang^2^, Deepak Dugar^3^, & José L. Avalos^1,2,4,5*^

1. Department of Chemical and Biological Engineering, Princeton University, Princeton, NJ 08544, USA.
2. Department of Molecular Biology, Princeton University, Princeton, NJ 08544, USA.
3. Visolis, Inc., 1488 Zephyr Ave. Hayward, CA 94544.
4. The Andlinger Center for Energy and the Environment, Princeton University, Princeton, NJ 08544, USA.
5. High Meadows Environmental Institute, Princeton University, Princeton, NJ 08544, USA.

^†^ These authors made equal contributions

Corresponding author address – 101 Hoyt Laboratory, 25 William Street Princeton, NJ 08544.

^*^Correspondence: javalos@princeton.edu

Key words – Metabolic engineering, mevalonate, acetyl-CoA, *Saccharomyces cerevisiae*, *ERG9,* pantothenate

**Supplementary Figure 1**. Time courses of 7-day fermentations with the wild-type strain (CEN.PK-1C) transformed with an empty 2µ plasmid (YLG6), or transformed (SIY) with a 2µ plasmid containing the mevalonate cassette, including *Ef-mvaE*, *Ef-mvaS* and *Se-acs^L641P^* (SIY2), compared to the *adh1Δgpd1Δ* double deletion background strain transformed with the same mevalonate cassette (JCY7) , showing cell growth (A), mevalonate production (B), glucose consumption (C), glycerol production (D), and ethanol production (E). Cell cultures were sampled every 24 hours for the first five days of fermentation, as well as at the end of the 7-day course. Error bars represent one standard deviation from three biologically independent replicates. *** P < 0.001.


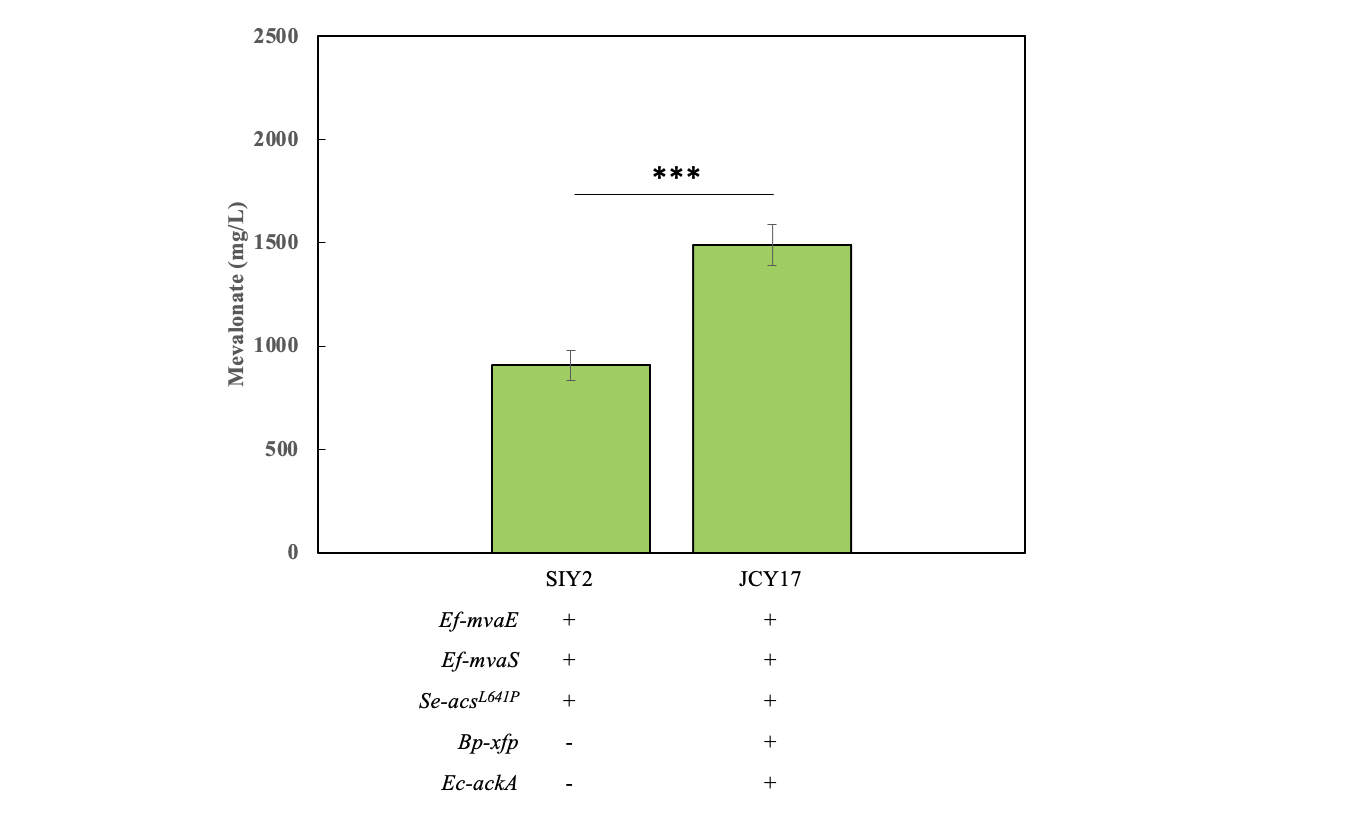


**Supplementary Figure 2**. Activity of *Bp-xfp* and *Ec-ackA* in *Saccharomyces cerevisiae*. The mevalonate cassette (containing *Ef-mvaE*, *Ef-mvaS* and *Se-acs^L641P^*) was introduced in a 2µ plasmid using *URA3* selection marker, while in the bifid shunt enzymes were supplied on a second 2µ plasmid using the LEU2 selection marker. Mevalonate production in 7-day fermentations starting at low cell density, with their respective selection condition (-uracil for a single plasmid, -uracil, -leucine for the two-plasmid strain). Error bars represent one standard deviation from three biologically independent replicates. *** P < 0.001.


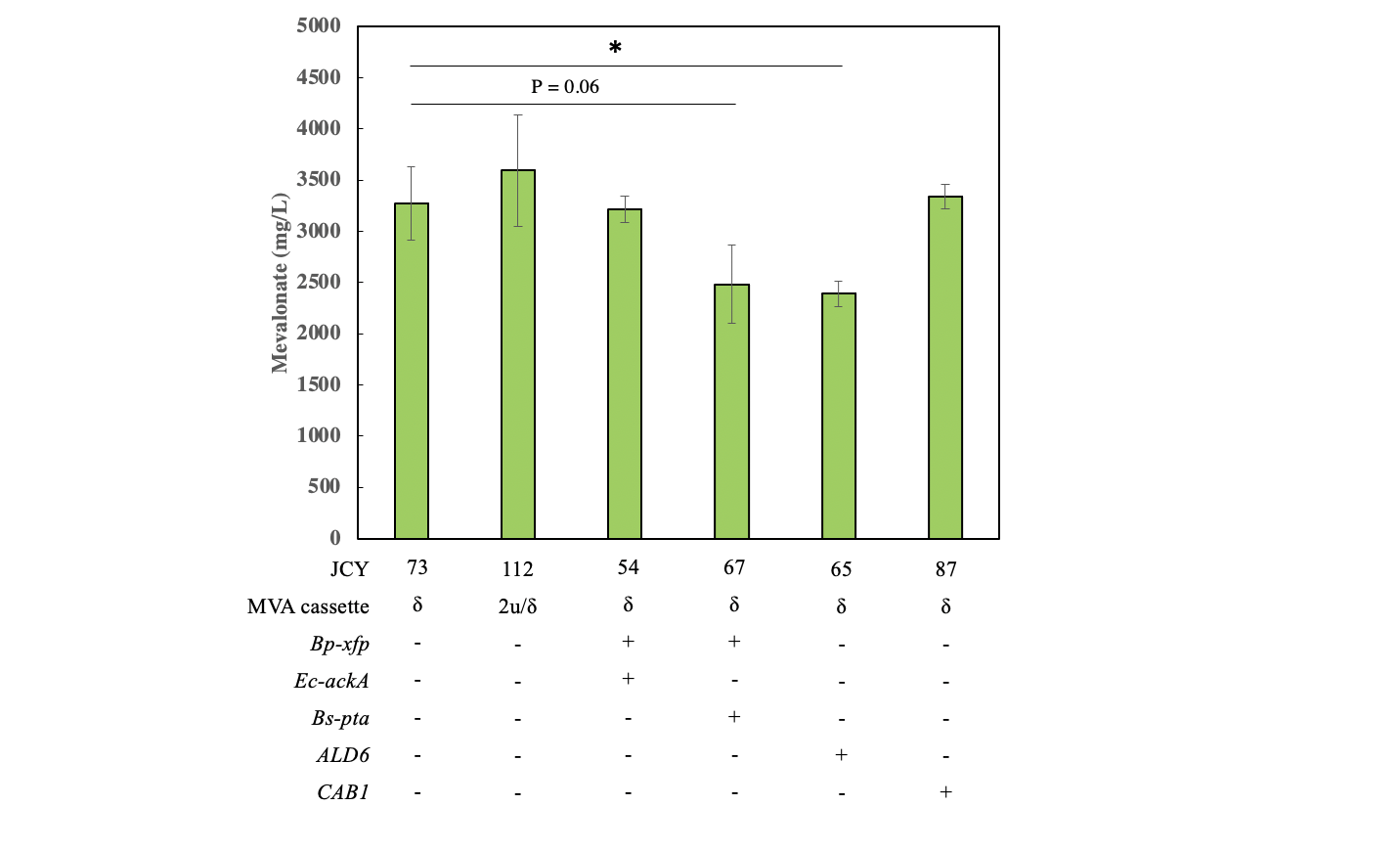


**Supplementary Figure 3:** Effect on mevalonate production of bifid shunt enzymes, or overexpression of *ALD6* or *CAB1* in strains with *ERG9* repression. All strains contain the methionine repressible *pMET3-ERG9* construct, which was inhibited by addition of 2 mM methionine at the beginning of the 7-day fermentation (see methods). JCY73 is a control strain containing an empty 2µ plasmid, while in all other strains additional pathway components were expressed with a 2µ plasmid. Error bars represent one standard deviation from three biologically independent replicates. *P < 0.005.


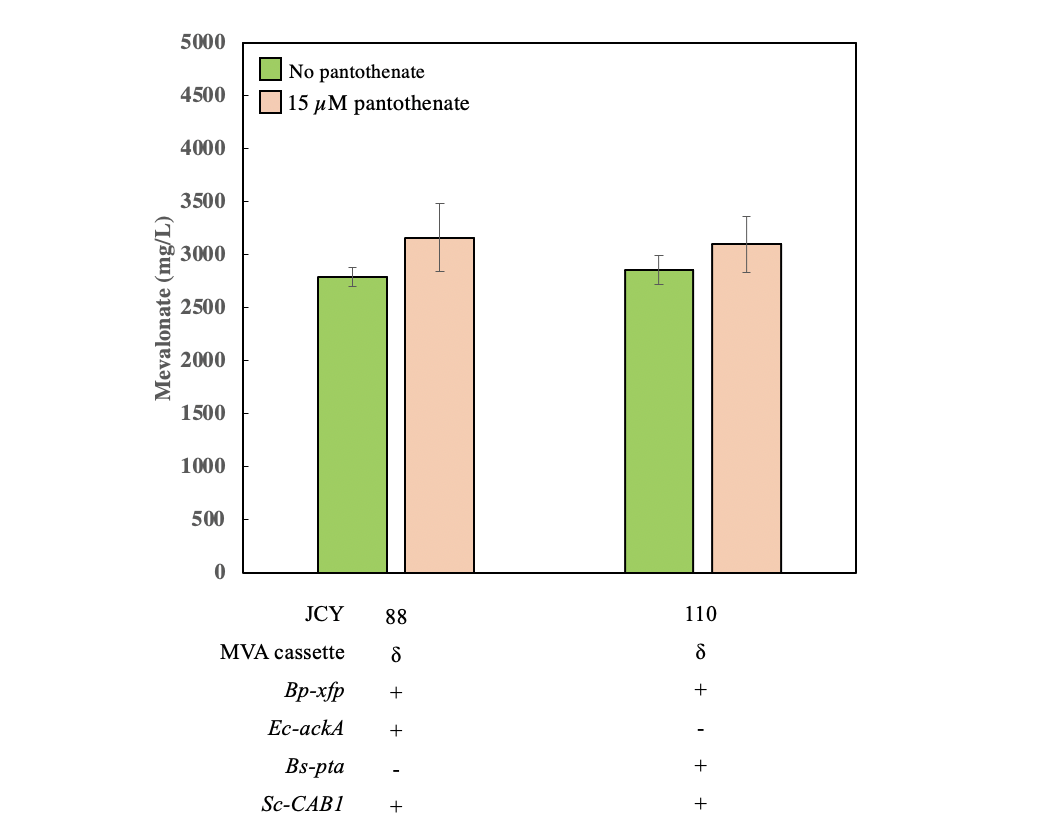


**Supplementary Figure 4:** Mevalonate production in strains with bifid-shunt enzymes, *CAB1* overexpression and *ERG*9 repression, with or without pantothenate supplementation. Mevalonate titers with 2 mM methionine added at the beginning of fermentation in cell lines expressing the acetate kinase route (JCY88) or the phosphotransacetylase route (JCY110) of the bifid-shunt in combination with *CAB1* expressed from a single 2µ plasmid. Experiments were conducted in 7-day fermentations starting at low cell density with/without 15 µM pantothenate. Error bars represent one standard deviation from three biologically independent replicates.

**Supplementary Sequence 1**. The repair template, CRISPR-*ERG9*-RT, is composed of three DNA segments - a truncated 3’ DNA segment of *PTH1* followed by P*_ERG9_*, *HIS3* marker cassette, and a truncated 5’ DNA segment of *ERG9* placed behind the P*_MET3_.* The full DNA sequence is provided below: no highlight – native sequence flanking site of interest, blue text – native *ERG9* promoter, yellow text – full *HIS3* marker, purple text – pMET3 promoter, red text – *ERG9* ORF segment. Lowercase bold letters in the *ERG9* ORF represent the silent mutations introduced into the repair construct to prevent recognition of the PAM-site by CAS9 in correct integrants.

5’-

GGTCATAATGGGCTGCGAAGCCTGCTAAAATGCAGTGGAGGCCGTGTACCCTTTGCCAAATTGGCTATTGGAATCGGCAGAGAACCTGGGTCCCGTTCTAGAGACCCTGCGAGCGTGTCCCGGTGGGTTCTGGGAGCTCTAACTCCGCAGGAACTACAAACCTTGCTTACACAGAGTGAACCTGCTGCCTGGCGTGCTCTGACTCAGTACATTTCATAGCCCATCTTCAACAACAATACCGACTTACCATCCTATTTGCTTTGCCCTTTTTCTTTTCCACTGCACTTTGCATCGGAAGGCGTTATCGGTTTTGGGTTTAGTGCCTAAACGAGCAGCGAGAACACGACCACGGGCTATATAAATGGAAAGTTAGGACAGGGGCAAAGAATAAGAGCACAGAAGAAGAGAAAAGACGAAGAGCAGAAGCGGAAAACGTATACACGTCACATATCACACACACACACGTTTTAAGAGCTTGGTGAGCGCTAGGAGTCACTGCCAGGTATCGTTTGAACACGGCATTAGTCAGGGAAGTCATAACACAGTCCTTTCCCGCAATTTTCTTTTTCTATTACTCTTGGCCTCCTCTAGTACACTCTATATTTTTTTATGCCTCGGTAATGATTTTCATTTTTTTTTTTCCACCTAGCGGATGACTCTTTTTTTTTCTTAGCGATTGGCATTATCACATAATGAATTATACATTATATAAAGTAATGTGATTTCTTCGAAGAATATACTAAAAAATGAGCAGGCAAGATAAACGAAGGCAAAGATGACAGAGCAGAAAGCCCTAGTAAAGCGTATTACAAATGAAACCAAGATTCAGATTGCGATCTCTTTAAAGGGTGGTCCCCTAGCGATAGAGCACTCGATCTTCCCAGAAAAAGAGGCAGAAGCAGTAGCAGAACAGGCCACACAATCGCAAGTGATTAACGTCCACACAGGTATAGGGTTTCTGGACCATATGATACATGCTCTGGCCAAGCATTCCGGCTGGTCGCTAATCGTTGAGTGCATTGGTGACTTACACATAGACGACCATCACACCACTGAAGACTGCGGGATTGCTCTCGGTCAAGCTTTTAAAGAGGCCCTAGGGGCCGTGCGTGGAGTAAAAAGGTTTGGATCAGGATTTGCGCCTTTGGATGAGGCACTTTCCAGAGCGGTGGTAGATCTTTCGAACAGGCCGTACGCAGTTGTCGAACTTGGTTTGCAAAGGGAGAAAGTAGGAGATCTCTCTTGCGAGATGATCCCGCATTTTCTTGAAAGCTTTGCAGAGGCTAGCAGAATTACCCTCCACGTTGATTGTCTGCGAGGCAAGAATGATCATCACCGTAGTGAGAGTGCGTTCAAGGCTCTTGCGGTTGCCATAAGAGAAGCCACCTCGCCCAATGGTACCAACGATGTTCCCTCCACCAAAGGTGTTCTTATGTAGTGACACCGATTATTTAAAGCTGCAGCATACGATATATATACATGTGTATATATGTATACCTATGAATGTCAGTAAGTATGTATACGAACAGTATGATACTGAAGATGACAAGGTAATGCATCATTCTATACGTGTCATTCTGAACGAGGCGCGCTTTCCTTTTTTCTTTTTGCTTTTTCTTTTTTTTTCTCTTGAACTCGATTTAGTACTAACAGAGACTTTTGTCACAACTACATATAAGTGTACAAATATAGTACAGATATGACACACTTGTAGCGCCAACGCGCATCCTACGGATTGCTGACAGAAAAAAAGGTCACGTGACCAGAAAAGTCACGTGTAATTTTGTAACTCACCGCATTCTAGCGGTCCCTGTCGTGCACACTGCACTCAACACCATAAACCTTAGCAACCTCCAAAGGAAATCACCGTATAACAAAGCCACAGTTTTACAACTTAGTCTCTTATGAAGTTACTTACCAATGAGAAATAGAGGCTCTTTCTCGAGAAATATGAATATGGATATATATATATATATATATATATATATATATATATATGTAAACTTGGTTCTTTTTTAGCTTGTGATCTCTAGCTTGGGTCTCTCTCTGTCGTAACAGTTGTGATATCGTTTCTTAACAATTGAAAAGGAACTAAGAAAGTATAATAATAACAAGAATAAAGTATAATTAACATGGGAAAGCTATTACAATTGGCATTGCATCCGGTCGAGATGAAGGCAGCTTTGAAGCTGAAGTTTTGCAGAACACCGCTATTCTCCATCTATGATCAGTCCACGTCTCCATATCTCTTGCACTGTTTCGAACTGTTGAACTTGAC**a**TC**a**AGATCGTTTGCTGCTGTGATCAGAGAGCTGCATCCAGAATTGAGAAACTGTGTTACTCTCTTTTATTTGATTTTAAGGGCTTTGGATACCATCGAAGACGATATGTCCATCGAACACGATTTGAAAATTGACTTGTTGCGTCACTTCCACGAGAAATTGTTGTTAACTAAATGGAGTTTCGACGGAAATGCCCCCGATGTGAAGGACAGAGCCGTTTTGACAGATTTCGAATCGATTCTTATTGAATTCCACAAATTGAAACCAGAATATCAAGAAGTCATCAAGGAGATCACCGAGAAAATGGGTAATGGTATGGCCGACTACATCTTgGATGAAAATTACAACTTGAATGGGTTGCAAACCGTCCACGACTACGACGTGTACTGTCACTACGTAGCTGGTTTGGTCGGTGATGGTTTGACCCGTTTGATTGTCATTGCCAAGTTTGCCAACGAATCTTTG-3’
